# Uptake Transporters of the SLC21, SLC22A, and SLC15A Families in Anticancer Therapy—Modulators of Cellular Entry or Pharmacokinetics?

**DOI:** 10.3390/cancers12082263

**Published:** 2020-08-12

**Authors:** Karin Brecht, Anima Magdalena Schäfer, Henriette E. Meyer zu Schwabedissen

**Affiliations:** Biopharmacy, Department of Pharmaceutical Sciences, University of Basel, 4056 Basel, Switzerland; anima.schaefer@unibas.ch (A.M.S.); h.meyerzuschwabedissen@unibas.ch (H.E.M.z.S.)

**Keywords:** solute carrier transporters, genetic variants, cancer, chemotherapy, tumor

## Abstract

Solute carrier transporters comprise a large family of uptake transporters involved in the transmembrane transport of a wide array of endogenous substrates such as hormones, nutrients, and metabolites as well as of clinically important drugs. Several cancer therapeutics, ranging from chemotherapeutics such as topoisomerase inhibitors, DNA-intercalating drugs, and microtubule binders to targeted therapeutics such as tyrosine kinase inhibitors are substrates of solute carrier (SLC) transporters. Given that SLC transporters are expressed both in organs pivotal to drug absorption, distribution, metabolism, and elimination and in tumors, these transporters constitute determinants of cellular drug accumulation influencing intracellular drug concentration required for efficacy of the cancer treatment in tumor cells. In this review, we explore the current understanding of members of three SLC families, namely *SLC21* (organic anion transporting polypeptides, OATPs), *SLC22A* (organic cation transporters, OCTs; organic cation/carnitine transporters, OCTNs; and organic anion transporters OATs), and *SLC15A* (peptide transporters, PEPTs) in the etiology of cancer, in transport of chemotherapeutic drugs, and their influence on efficacy or toxicity of pharmacotherapy. We further explore the idea to exploit the function of SLC transporters to enhance cancer cell accumulation of chemotherapeutics, which would be expected to reduce toxic side effects in healthy tissue and to improve efficacy.

## 1. Introduction

In 2018, the International Agency for Research on Cancer estimated that 18.1 million new cancer cases occurred worldwide. With estimated 9.6 million deaths in the same year, cancer was the second leading cause of death globally, with approximately 70% of cancer deaths occurring in low and middle-income countries [1]. On average, there is about a 20% risk of getting cancer before age 75 and a 10% risk of dying from it [2].

To date, surgical removal of the tumor, radiotherapy, and chemotherapy given alone or in combination constitute the main pillars in the management of cancer. Besides the “classical” chemotherapeutic drugs that inhibit DNA synthesis and cell division and induce apoptosis, drugs that target tumor-specific signaling pathways such as the tyrosine kinase inhibitors have been developed and successfully included in cancer treatment regimens [3]. Novel therapeutic strategies including gene therapy and immunotherapy, however, are extremely costly and technically sophisticated, and success rates are rather limited [4]. Thus, chemotherapy with small molecules is still an important treatment modality with broad application. The success of any therapeutic intervention depends on the pharmacokinetics (PK) of the drug. The handling of a drug in the human organism depends on the ADME processes, where each step absorption, distribution, metabolism, and excretion involves movement across a membrane. This transmembrane transport is assumed to be determined by the coordinate work of influx transporters and efflux transporters. These membrane proteins are expressed not only in organs pivotal to drug handling such as the intestine, liver, and kidney but also in cancer cells. Thus, drug transporters are determinants of cellular drug accumulation influencing intracellular drug concentration required for efficacy of the cancer treatment in tumor cells.

In general, drug transporters are classified into ATP binding cassette (ABC) transporters and solute carrier (SLC) transporters. ABC transporters are drug efflux transporters such as P-glycoprotein (P-gp/ABCB1), the multidrug resistance-associated proteins (MRPs/ABCCs), and breast cancer resistance protein (BCRP/ABCG2) [5]. Uptake transporters primarily belong to the SLC transporter superfamily, which comprises 65 gene families with 458 transporter genes in humans described to date [6,7]. SLC transporters are expressed not only in all vital organs such as the liver, kidney, intestine, brain, heart, and lung but also in several tumor types. They facilitate the transport of a broad range of endogenous nutrients, neurotransmitters, hormones, pharmaceutical compounds, and toxins [8].

In this review, we summarize the current understanding of SLC transporters in the etiology of cancer, in transport of chemotherapeutic drugs, and their influence on efficacy or toxicity of pharmacotherapy. We further explore the idea to exploit the function of SLC transporters to enhance cancer cell accumulation of chemotherapeutics which would be expected to reduce toxic side effects in healthy tissue and to improve efficacy. We focus on three SLC families considered to play a particularly important role in cellular uptake of pharmaceutical agents, i.e., the *SLC21* (organic anion transporting polypeptides, OATPs), *SLC22A* (organic cation transporters, OCTs; organic cation/carnitine transporters, OCTNs; and organic anion transporters, OATs), and *SLC15A* (peptide transporters, PEPTs).

## 2. Organic Anion Transporting Polypeptides in Anticancer Therapy

One family of drug transporters widely studied in pharmacology are the organic anion transporting polypeptides (OATPs). These membrane proteins are expressed in a variety of organs and transport a broad range of endogenous compounds, pharmacological drugs, and toxins rendering OATPs an important determinant in drug absorption, distribution, metabolism, and elimination. Eleven OATPs have been identified so far and are encoded by the *SLCO* (formerly *SLC21*) genes. The human OATPs are classified into six families with the OATP1 family subsuming four members (OATP1A2, OATP1B1, OATP1B3, and OATP1C1) and the OATP2 family with so far two members identified (OATP2A1 and OATP2B1). The remaining families constitute only one member each, and these are OATP3A1, OATP4A1, OATP5A1, and OATP6A1 [9]. Following, we will focus on the role of OATP1B1 and 1B3, OATP1A2, and OATP2B1 in cancer etiology and treatment. These transporters have been extensively investigated with respect to (i) transport of steroid hormones and their precursors in hormone-dependent cancers driving the proliferation of cancer cells and (ii) transport of antineoplastic drugs.

### 2.1. OATP1B Transporters

The OATP1B family of organic anion transporting polypeptides is the most famous in terms of uptake transporters influencing the in vivo handling of drugs. This subfamily consists of three members namely OATP1B1, OATP1B3, and OATP1B3-1B7 readthrough [10,11,12]. These transporters are highly abundant in the liver, where they have a crucial role in drug metabolism and elimination. While OATP1B1 and OATP1B3 are expressed at the sinusoidal membrane of hepatocytes, OATP1B3-1B7 was found to be localized to the smooth endoplasmic reticulum of hepatocytes and enterocytes [9]. In particular, OATP1B3 has been detected in several extrahepatic malignant tissues such as breast, prostate, colorectal, lung, and pancreas tumors, with details excellently summarized by Obaidat et al. [13].

Especially for OATP1B1, it is known that frequently occurring function-impairing polymorphisms translate into reduced hepatic uptake and therefore enhanced systemic exposure with substrate drugs [14]. This has been validated in multiple studies on statins, which could nearly be considered probe drugs of hepatic OATP1B function [15,16]. Consequently, most of the studies on OATP1B1 and OATP1B3 in cancer therapy also investigate the impact of these hepatic transporters on systemic exposure. The spectrum of anticancer compounds known to be substrates of OATP1B1 and/or OATP1B3 is broad, including atrasentan, sorafenib-glucuronide (SG), SN-38, docetaxel, methotrexate (MTX), paclitaxel, and doxorubicin [17,18,19,20].

One class of anticancer drugs investigated for the influence of OATP1B transporters are tyrosine kinase inhibitors. In this context, we want to highlight the findings on sorafenib-glucuronide, which were the basis of further studies, elucidating the function of the hepatic uptake transporters in a pharmacological concept of “hepatocyte-hopping”, which was first proposed in the context of hepatic bilirubin metabolism [21]. Zimmerman et al. [18] were able to show that sorafenib-glucuronide, a major inactive metabolite of sorafenib, is a substrate of OATP1B1 and OATP1B3 in vitro. Moreover, the authors report in vivo data applying the Oatp1a/1b deficient or the humanized OATP1B1 and OATP1B3 mouse model. Here, they observed that animals lacking the Oatp1a/1b-gene locus exhibited significantly increased sorafenib-glucuronide plasma levels, which were restored after transgenic expression of human OATP1B1 or OATP1B3, respectively. No influence on the parent compound was observed [18]. In following animal studies, the assumed “hepatocyte-hopping” mechanism was further consolidated, where SG was formed in one hepatocyte, extruded into systemic circulation by efflux transporters, and then depended on sinusoidal uptake by OATP1B transporters in the next hepatocytes [22]. This explains the finding of hepatically formed metabolites being influenced by the activity of transporters previously assumed to facilitate the step prior to enzymatic modification [23]. Accordingly, in humans, receiving sorafenib and rifampicin, a potent inhibitor of the organic anion excretion route involving multiple transporters (such as OATPs and ABC transporters), there was a significant increase in SG when rifampicin was co-administered [24]. Even if there are currently no studies on the influence of genetic polymorphisms of OATP1B1 or OATP1B3 on SG pharmacokinetics, Bins et al. [25] observed an association between the *SLCO1B1* variants rs2306283 (c.388A>G, p.N130D) and rs4149056 (c.521C>T, p.V174A), and diarrhea and thrombocytopenia. Briefly, the 521T allele and 388A allele were shown to be significantly associated with increased odds of thrombocytopenia or diarrhea, respectively, but not with concomitant effects on survival [25] (Table 1). In the context of transporters influencing therapy response to sorafenib treatment, the organic cation transporters should be mentioned. Although there are contradictory in vitro data on OCT1 (*SLC22A1*) influencing its cellular accumulation [26,27,28,29], clinical data link sorafenib to the transporter. Low intratumoral OCT1 expression was reported to be associated with a worse survival of patients with hepatocellular carcinoma (HCC), while presence of the transporter at the plasma membrane was beneficial for therapy response [30,31] (Table 2). Notably, compared to nonmalignant transformed hepatocytes, OCT1 is downregulated in HCC, which has been explained by hypermethylation of the *SLC22A1* promotor region, and in vivo models showed that downregulation is linked to decreased sorafenib uptake in the tumor [32,33]. Moreover, genetic variants of OCT1 were shown to have an impact on the transporter function and hence on cellular sorafenib uptake [28]. Recently, PEPT2 (*SLC15A1*) has also been associated with response to sorafenib. The non-synonymous genetic variant rs2257212 (*SLC15A1* c.1048C>T, p.L350F) was associated with prolonged progression-free survival (PFS) in patients with unresectable hepatocellular carcinoma (HCC) treated with sorafenib. Here, patients with the 1048T/T or C/T genotypes displayed a significantly longer PFS than did patients with C/C genotypes [34]. In line with this finding, this group observed that the HCC cell line SNU182 possessing the T/T genotype was more sensitive to sorafenib than Hep3B carrying the 1048C/C genotype. The indirect character of these in vitro results and given the rather atypical molecular properties of sorafenib, surely more studies are needed to prove the role of PEPT2 in sorafenib transport [35].

Another compound studied in the context of OATP1B function in liver is the topoisomerase I inhibitor and semisynthetic camptothecin analogue irinotecan. Irinotecan is a prodrug which is readily and mainly converted to its active metabolite SN-38, which is further metabolized to SN-38 glucuronide. Irinotecan therapy is hampered by severe toxicity with the risk for life-threatening neutropenia or diarrhea, which is primarily caused by the active metabolite SN-38 [36]. Iusuf et al. [19] demonstrated that plasma concentration of SN-38 but not the parent compound increased in Oatp1a/1b knockout as compared to wildtype mice. This phenotype was reverted in mice humanized for OATP1B1 or OATP1B3, suggesting a direct role of OATP1B transporters in SN-38 disposition [19]. Testing the influence of the variants *SLCO1B1* c.521T>C, p.Val174Ala (rs4149056), *SLCO1B1* c.388A>G, p.Asn130Asp (rs2306283), and −11187GG (rs4149015) in clinical trials revealed an impact on systemic SN-38 exposure, adverse effects, or efficacy [37,38]. In detail, carriers of the low function c.521C allele had increased exposure and a higher risk for grade 4 neutropenia as compared to carriers of the wildtype allele [37]. In the same study, a positive correlation between the c.388GG genotype and longer PFS as well as an increased incidence of grade 3 diarrhea has been observed. Hepatic uptake transporters other than OATP1B may also be involved in the handling of irinotecan or its metabolites [39]. Fujita et al. [39] elucidated that SN-38 was a substrate of OATP2B1 when OATP2B1 was overexpressed in *Xenopus laevi* oocytes. Concomitant treatment with baicalin, apple juice, or cefixime dose-dependently inhibited OATP2B1-mediated SN-38 transport. In the same study, they demonstrated that mice orally administered SN-38 (10 mg/kg) for five consecutive days showed signs of gastrointestinal toxicity as presented by shortening of the intestinal villus. SN-38-treated mice concurrently receiving apple juice had slightly but significantly less gastrointestinal side effects as compared to mice receiving SN-38 only [39].

Genetic variability of OATP1B transporters was also applied to investigate the role in pharmacokinetics and/or efficacy of other antitumor drugs. Briefly, Drenberg et al. [51] investigated inherited genetic variants and clinical outcomes in children with acute myeloid leukemia (AML) receiving a chemotherapeutic combination of cytarabine, daunorubicin, etoposide, and mitoxantrone. They used the Affymetrix DMET Plus^™^ platform genotyping 225 genes with contribution to ADME and found that the synonymous single nucleotide polymorphism (SNP) rs2291075 in *SLCO1B1* (c.597C>T) was significantly associated with both event-free survival and overall survival (patients carrying homozygous (T/T) presenting improved survival, whereas patients with (C/C) had the most unfavorable outcome).

In vitro experiments overexpressing different OATP1B1 genetic variants in HEK cells confirmed that *SLCO1B1* haplotypes including the rs2291075 variant exhibit reduced transport activity of cytarabine, daunorubicin, etoposide, or mitoxantrone. In line with this, Oatp1b2 knockout mice showed a decrease in liver accumulation and an increase in plasma levels of daunorubicin, etoposide, and mitoxantrone as compared to wildtype mice. These findings clearly suggest that OATP1B1 contributes to the PK and pharmacodynamics (PD) of these anticancer drugs [51]. The number of polymorphisms reported for the *SLCO1B1* and *SLCO1B3* genes with respect to MTX, doxorubicin, and docetaxel are considerable. In general, characterization of OATP1B1 and OATP1B3 function using knockout and/or humanized mice models and investigation of in vitro transport capacities using cell lines overexpressing genetic variants of the transporters have significantly contributed to the understanding of potential variabilities in PK, PD, and safety [59,60,61,62]. However, clinical trials aiming to clarify the significance of *SLCO1B* genetic variants in MTX, doxorubicin, paclitaxel, and docetaxel disposition and efficacy are controversial [63,64,65], which may reflect ethnic-specific differences, a limited number of patients included in these studies, or neglecting the linkage effect of the gene regions. In two recent reviews by Schulte et al. [66] and Choudhuri et al. [67], respectively, known genetic polymorphism in the *SLCO1B* genes have been comprehensively summarized in this perspective.

The abovementioned pharmacogenetic studies mostly focused on OATP1B1. Even if there are naturally occurring polymorphisms of OATP1B3, which influence expression and transport function in vitro, there is currently only limited evidence from clinical studies showing the impact of OATP1B3 on systemic pharmacokinetics of substrate drugs [68]. However, Hamada et al. [69] tested cell lines of different tumor entities for the most frequent polymorphisms, namely *SLCO1B3* rs4149117 (c.334T>G, p.112S>A) and *SLCO1B3* rs731135 (c.699G>A, p.M233I), showing that the linkage of these polymorphisms is conserved during malignant transformation. However, in accordance with previous findings by Nies et al. [70] in human liver, the genetic variants were not predictive for the amount of transporter expressed in the cells [69,70]. In prostate cancer patients, the authors observed a longer survival for those individuals carrying the homozygous variant-haplotype, whereby linking their in vitro result on impaired testosterone transport by the variant protein to the observation of enhanced presence of OATP1B3 in prostate cancer cells. The authors hypothesized that the enhanced uptake of androgens in patients carrying the wildtype haplotype may accelerate androgen independence [69].

Several cancer cells were found to express variant forms of OATP1B3, referred to as cancer-type OATP1B3 (ct-OATP1B3). Two groups namely Thakkar et al. [71] and Imai et al. [72] independently reported ct-OATP1B3 V1 to be the prevalent cancer-specific form of OATP1B3 in human colon and pancreatic cancers and different tumor cell lines, respectively. As compared to the wildtype form of OATP1B3, ct-OATP1B3 V1 lacks exons 1 and 2 but contains an alternative exon 2a and an alternative translation start codon in exon 3. Hence, the ct-OATP1B3 V1 protein presents with only 674 amino acids as compared to 702 amino acids for OATP1B3. There is inconsistent data, however, regarding the localization and transport activity of ct-OATP1B3 V1. While Imai et al. [72] reported ct-OATP1B3 V1 to be localized to the plasma membrane and to be a functional transporter when expressed in HEK293 cells, Thakkar et al. [71] showed predominant localization in the cytoplasm and, consequently, a significant reduction in transport function as compared to OATP1B3 [71,72]. Nagai et al. [73] reported a slightly different ct-OATP1B3 variant, referred to as ct-OATP1B3 C. ct-OATP1B3 C has its translation initiation start in exon 3, is 655 amino acids long, and is predominantly detected in human lung and colon cancer tissues. This group detected the absence of transport activity in ct-OATP1B3 C overexpressing HCT116 cells [74]. In general, functional ct-OATP1B3—if really cancer specific—may have the potential to serve as an active targeting mechanism for chemotherapeutics. In this sense, discovering compounds with specificity for ct-OATP1B3 would clearly constitute a valuable target of chemotherapeutics in cancer cells while sparing healthy cells. Hence, specificity for a single transporter is currently rather a rare phenomenon, but after verifying the localization and functionality of ct-OATP1B3 in cancer cells, it remains to be tested whether this protein could be exploited as an active targeting mechanism.

### 2.2. OATP1A2

The substrate spectrum of OATP1A2 ranges from endogenous compounds such as bile acids, steroid hormones and their conjugates (estrone 3-sulfate (E1S), estradiol 17β-D-glucuronide (E2G), and dehydroepiandrosterone sulfate (DHEAS)), prostaglandins, and thyroid hormones to various xenobiotics such as fexofenadine, ouabain and microcystin [75]. OATP1A2 expression was investigated in various human malignancies. In osteosarcoma, benign bone tumors, and bone metastases from primary tumors of different origin, *SLCO1A2* mRNA expression levels were variable, making OATP1A2 a rather weak prognostic marker in these tumor entities [76]. Colon cancer tissue and polyps displayed decreased expression of *SLCO1A2* mRNA levels as compared to healthy colon tissue [77].

The first type of cancer reported to be associated with an increased expression of OATP1A2 was breast cancer. Miki et al. [78] detected high levels of *SLCO1A2* mRNA in several breast carcinoma cell lines and in human cancer tissue biopsies but not in adjacent noncancerous breast tissues, adipose tissues, or stromal cells. In the same publication and in the work of Meyer zu Schwabedissen et al. [79], an association between the expression level and the function of OATP1A2 and nuclear pregnane X receptor (PXR) was observed [78,79]. Using T47-D breast carcinoma cells, the PXR inducer rifampicin resulted in a significant increase in OATP1A2 expression and a concomitant accumulation of intracellular E1S, which was correlated with promoted breast cancer cell proliferation in vitro [79]. Even if there is a mechanistic link between OATP1A2, PXR, and steroid uptake, neither the risk to develop breast cancer nor tumor characteristics were affected when testing genetic variants in a human breast cancer cohort [80]. However, further following the path of enhanced OATP1A2 expression as a modifier of breast cancer, Banerjee et al. [81] found enhanced expression of OATP1A2 in MCF-7 cells compared to nontumorous MCF-10A cells. The same group demonstrated that MCF-7 tumor-bearing xenograft mice displayed a significant uptake of E1S into the tumor graft [82]. Early imaging studies by Kenady et al. [83] and by Bénard et al. [84] demonstrated accumulation of labeled estradiol in breast carcinoma. The authors concluded that estradiol-coupled radiotracers such as ^18^F-fluoroestradiol (^18^F-FES) or ^123^I-estradiol (^123^I-E) might constitute suitable molecules for diagnostic imaging of estradiol-dependent cancers especially for tumors displaying mutations or loss of the estrogen receptor [83,84]. Further studies are needed to corroborate whether ^18^F-FES and ^123^I-E indeed constitute suitable tracers for breast carcinoma, and it would be interesting to investigate whether OATP1A2 is a determinant for uptake into tumor cells.

In view of breast cancer therapy and OATP1A2, there are reports linking high expression of OATP1A2 and OCT6 in triple-negative breast cancer to therapy response. Indeed, in a small retrospective study, both transporters were independent predictors of a good response to doxorubicin- and taxane-based chemotherapy [58]. Similar results were obtained for docetaxel, a semisynthetic analogue of paclitaxel and microtubule inhibitor. In another breast cancer study, it was found that the T-T haplotype of two identified SNPs located in intron 1 of the *SLCO1A2* gene, rs4762699 and rs2857468, were highly associated with the risk of life-threatening febrile neutropenia in breast cancer patients treated with docetaxel (along with doxorubicin) [50]. Interestingly, doxorubicin was identified as a substrate of OCT6 and an influence on doxorubicin pharmacokinetic was seen in Asian breast cancer patients carrying a genetic variant showing a trend to higher exposure levels of doxorubicin [40,85].

Staying within the field of hormone-dependent tumors, OATP1A2 has also been reported to be expressed in the human androgen receptor-positive prostate cancer cell lines, LNCaP and 22Rv1. Here, the transporter has been assumed to facilitate the uptake of DHEAS (which is mainly produced in the adrenal cortex), enhancing cancer cell growth under otherwise androgen-depleted (AD) conditions. This observation led to the speculation that high in vivo DHEAS serum levels may display an alternative source of androgen, which allows prostate cancer cells to proliferate despite AD therapy (ADT). Moreover, this suggests that pharmacological inhibition of an OATP1A2-mediated influx of DHEAS in combination with ADT might provide a clinical benefit for patients with prostate cancer [86]. It seems noteworthy at this point that Wright et al. [87] did not find significant differences in the expression of *SLCO1A2* comparing matched benign and cancer prostate samples from eight untreated patients. In addition, one should generally consider the expression of OATP1A2 in healthy, nonmalignant tissue such as testis, renal tubule cells, and liver cholangiocytes [88,89,90]. Also, the brain/the blood–brain barrier (BBB) expression of OATP1A2 has been independently reported by different groups, and we observed OATP1A2 in glial cells, even though there are reports showing OATP1A2 protein levels to be below the lower limit of quantitation applying quantitative targeted proteomics [43,91,92,93,94,95]. The expression in these organs might give rise to unwanted effects associated with inhibition of DHEAS transport.

Anticancer drugs with frequent application in therapeutic regimens and shown to be substrates of OATP1A2 are imatinib and MTX [96,97,98]. Imatinib is an approved inhibitor of BCR-ABL, and KIT tyrosine kinase is used in the treatment of Philadelphia chromosome-positive chronic myeloid leukemia (CML) and KIT-positive gastrointestinal stromal tumors (GIST), respectively [43,96]. Testing inherent non-synonymous polymorphisms of OCT1, OCTN1, and OATP1A2, it was found that a combination of SNPs in these transporters is associated with major and complete molecular response to imatinib therapy [44], suggesting the potential of genetic association studies to elucidate the role of OATP1A2 in imatinib therapy. However, testing the influence of the exonic genetic variant rs11568563 (c.516A>C, p.E172D, OATP1A2*3), which was associated with a complete lack of imatinib transport in vitro, Eechoute et al. [43] did not find significant changes in imatinib plasma levels in patients carrying this variant. Moreover, coadministration of rosuvastatin in this case used as an inhibitor of OATP1A2 (even if known to interact with multiple OATPs) did not affect imatinib plasma concentrations in cancer patients [43]. Patients with the *SLCO1A2* -361GG (rs3764043) genotype had a significantly lower imatinib clearance than those patients with the GA or AA genotype but without an impact on clinical response [45]. In PK studies in healthy men, imatinib showed rapid oral absorption, extensive oxidative metabolism in the liver, and excretion mainly via the biliary-fecal route [99]. Given that OATP1A2 is absent to minimally expressed in liver hepatocytes and the small intestine, the lack of association between *SLCO1A2* polymorphisms and imatinib plasma concentration is not unexpected. This further suggests that SLC transporters other than OATP1A2 are involved in ADME. There are reports about oncogenetic variants in the *SLC22A4* (OCTN1) and *SLC22A5* (OCTN2) genes to be linked to prolonged time to progression or optimal response to imatinib treatment in different cancers [46,100]. In the context of transporters influencing the in vivo handling of imatinib, the interaction with organic cation transporters should also be mentioned. Even if there are no consistent findings on OCT1 transporting the tyrosine kinase inhibitor, there are reports suggesting a link between OCT1 expression/activity and cellular uptake of imatinib [101,102]. In detail, testing isolated blood leukocytes of CML patients for imatinib uptake capacity in the presence or absence of the organic cation transporter inhibitor prazosin, the authors found that the size of the prazosin-inhibitable component is predictive for response to imatinib [103]. Furthermore, in patients with a large prazosin-inhibitable component, the clinical outcome was independent of imatinib dose, while in patients with a small prazosin-inhibitable component of imatinib accumulation, a high dose of imatinib resulted in superior molecular response [104]. Even though these results could be interpreted as evidence that OCT1 is involved, they have to be handled with reserve especially since there is no consistent findings on OCT1 mRNA expression in leukemic cells and its correlation to therapy response in CML patients [105]. In fact, an assay providing information about a patient-fitting dosage would be a great benefit. However, in the end, it will be difficult to separate the local from the systemic component especially since interindividual variability in imatinib pharmacokinetics after oral application was found to be considerable [106].

Anticancer drugs with frequent application in therapeutic regimens and shown to be substrates of OATP1A2 are imatinib and MTX [96,97,98]. Imatinib is an approved inhibitor of BCR-ABL, and KIT tyrosine kinase is used in the treatment of Philadelphia chromosome-positive chronic myeloid leukemia (CML) and KIT-positive gastrointestinal stromal tumors (GIST), respectively [43,96]. Testing inherent non-synonymous polymorphisms of OCT1, OCTN1, and OATP1A2, it was found that a combination of SNPs in these transporters is associated with major and complete molecular response to imatinib therapy [44], suggesting the potential of genetic association studies to elucidate the role of OATP1A2 in imatinib therapy. However, testing the influence of the exonic genetic variant rs11568563 (c.516A>C, p.E172D, OATP1A2*3), which was associated with a complete lack of imatinib transport in vitro, Eechoute et al. [43] did not find significant changes in imatinib plasma levels in patients carrying this variant. Moreover, coadministration of rosuvastatin in this case used as an inhibitor of OATP1A2 (even if known to interact with multiple OATPs) did not affect imatinib plasma concentrations in cancer patients [43]. Patients with the *SLCO1A2* -361GG (rs3764043) genotype had a significantly lower imatinib clearance than those patients with the GA or AA genotype but without an impact on clinical response [45]. In PK studies in healthy men, imatinib showed rapid oral absorption, extensive oxidative metabolism in the liver, and excretion mainly via the biliary-fecal route [99]. Given that OATP1A2 is absent to minimally expressed in liver hepatocytes and the small intestine, the lack of association between *SLCO1A2* polymorphisms and imatinib plasma concentration is not unexpected. This further suggests that SLC transporters other than OATP1A2 are involved in ADME. There are reports about oncogenetic variants in the *SLC22A4* (OCTN1) and *SLC22A5* (OCTN2) genes to be linked to prolonged time to progression or optimal response to imatinib treatment in different cancers [46,100]. In the context of transporters influencing the in vivo handling of imatinib, the interaction with organic cation transporters should also be mentioned. Even if there are no consistent findings on OCT1 transporting the tyrosine kinase inhibitor, there are reports suggesting a link between OCT1 expression/activity and cellular uptake of imatinib [101,102]. In detail, testing isolated blood leukocytes of CML patients for imatinib uptake capacity in the presence or absence of the organic cation transporter inhibitor prazosin, the authors found that the size of the prazosin-inhibitable component is predictive for response to imatinib [103]. Furthermore, in patients with a large prazosin-inhibitable component, the clinical outcome was independent of imatinib dose, while in patients with a small prazosin-inhibitable component of imatinib accumulation, a high dose of imatinib resulted in superior molecular response [104]. Even though these results could be interpreted as evidence that OCT1 is involved, they have to be handled with reserve especially since there is no consistent findings on OCT1 mRNA expression in leukemic cells and its correlation to therapy response in CML patients [105]. In fact, an assay providing information about a patient-fitting dosage would be a great benefit. However, in the end, it will be difficult to separate the local from the systemic component especially since interindividual variability in imatinib pharmacokinetics after oral application was found to be considerable [106].

Genetic studies in humans investigating OATP1A2 are certainly hampered by the low frequency of naturally occurring reduced function alleles [75,107]. Still, the influence of OATP1A2 on MTX PK has been investigated by Wang et al. [47] in a recent clinical trial including 60 rheumatoid arthritis patients. They found that only the *SLCO1A2* c.550G>A, p.E184K variant was associated with delayed MTX elimination and increased MTX-related toxicity. In contrast, three more genetic variants mentioned in the same study, c.553G>A, p.D185N, c.775A>C, p.V255I, and c.862G>A, p.T259P, which were functionally evaluated in HEK-293 cells and were found to have reduced expression and a concomitant decrease in MTX transport activity, were not associated with MTX PK or toxicity [48]. The role of OATP1A2 in MTX disposition has been shown by Van de Steeg et al. [108]. They reported that intravenous administration of MTX in Oatp1a/1b k.o. mice led to a significant increase in drug plasma levels with a concomitant reduction in liver accumulation (apparent by either reduced liver drug concentration or reduced liver-to-plasma ratios). Transgenic, hepato-specific expression of human OATP1A2 reverted the phenotype, at least partially [108]. In addition to OATP1A2, the PK of MTX depends on the folate transporters, proton-coupled folate transporter (PCFT, *SLC46A1*) and reduced folate carrier (RFC1, *SLC19A1*), OATs, OATP1B, and ABC transporters [97,109]. While PCFT is considered the major determinant in intestinal absorption, OAT1 and OAT3 (tubular secretion [110]) and OATP1A2 (distal tubular reabsorption) are believed to be involved in renal elimination. Around 80% of MTX is primarily excreted via the kidney in its unchanged form with transporter-mediated secretion prevailing over glomerular filtration. Notably, case reports of life-threatening kidney failure were reported after coadministration of MTX with probenecid, NSAIDs, or proton pump inhibitors, which supports an involvement of the OATs in tubular handling of MTX [111,112,113,114].

### 2.3. OATP2B1

OATP2B1 has a broad tissue distribution. Besides expression in organs vital to drug disposition such as the liver, kidney, and intestine, OATP2B1 is expressed in the heart, brain, placenta, spleen, ovary, kidney, lung, and skeletal and smooth muscle cells [115,116,117,118,119,120]. This member of the OATP family has a broad substrate specificity including several anticancer drugs such as abiraterone, erlotinib, etoposide, teniposide, and SN-38, to name a few of them [39,40,121,122]. OATP2B1 may, if differentially expressed, modify the handling of drugs in the microcompartment which consists of tumor cells and surrounding nonmalignant transformed tissue. We have previously described a similar concept for drug eluting stent (DES)-treated coronary arteries. Here, antiproliferative DES coating is used to inhibit proliferation of smooth muscle cells (SMCs), while it is intended to spare endothelial cells (ECs) from the antiproliferative effect to avoid an influence on endothelialization of the stent struts. Our data show that OATP2B1 is much higher expressed in the SMCs while only limited expression was observed in ECs. Moreover, in vitro data revealed enhanced antiproliferative efficacy of the OATP2B1 substrate teniposide in the cell-type expressing more of the transporter [120]. The combination of a drug delivery device with local release in the targeted microcompartment is unique for coronary artery stents. However, differential expression of OATP2B1 comparing malignant transformed and nonmalignant transformed tissue may still be of relevance in terms of tumor biology and drug efficacy.

In prostate cancer, the mRNA of *SLCO2B1* is one of six SLC transporters shown to be upregulated compared to nonmalignant transformed tissue. Whether this influences tumor development or progression is unclear. However, genetic studies revealed that patients somatically carrying the variant allele of *SLCO2B1* (SNP rs12422149, c.935G>A) have a higher risk of dying from prostate cancer. Similarly, carriers of the T allele in the *SLCO1B3* SNP rs4149117, c.334T>G had a 76% increased risk of prostate cancer-specific mortality. Considering the function of OATP2B1 as facilitator of the transmembrane transport of sulfated steroid metabolites such as DHEAS and E1S, it may be speculated that OATP2B1 is involved in the handling of steroids in prostate cells, influencing the intended ablation of testosterone as part of ADT [87,115,123]. However, this remains to be investigated.

**Figure 1:**
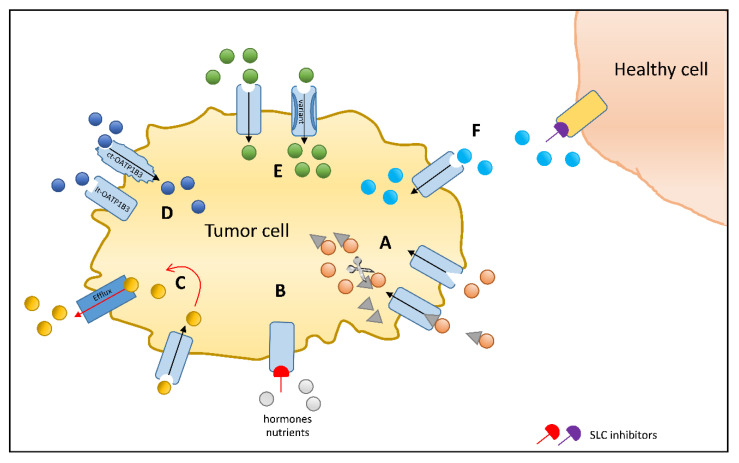
Solute carrier (SLC) uptake transporters as chemotherapeutic drug targets to increase efficacy or reduce toxicity: (**A**) Transporter-mediated accumulation of a chemotherapeutic agent conjugated to an endogenous substrate of an SLC transporter, which may be cleaved [125] or act as a conjugate [126]. (**B**) SLC transporter inhibition stops the accumulation of growth promoting factors [127]. (**C**) Coadministration of a chemotherapeutic drug and an inhibitor of efflux transporter [128]. (**D**) Ct-OATP1B3-specific chemotherapeutic substrates to enhance tumor cell accumulation sparing healthy tissue expressing lt-OATP1B3 [129]. (**E**) Identification of genetic variants exhibiting superior transport capacity as compared to the wildtype that could allow personalized medication and dose finding [41]. (**F**) Inhibition of an SLC transporter in healthy tissue such as the kidney in order to reduce toxic side effects [130].

In the context of prostate cancer, the studies on abiraterone and OATP2B1 should be mentioned. Abiraterone is an inhibitor of the multifunctional 17α-hydroxylase/17,20-lyase (CYP17). CYP17 mediates the conversion of pregnenolone and progesterone to 17α-hydroxypregnenolone and 17α-hydroxyprogesterone, which are then further converted to the weak androgens, dehydroepiandrosterone (DHEA) and androstenedione, respectively. Abiraterone was shown to be a substrate of OATP2B1 and OATP1B3 [40,124]. OATP2B1 as a modifier of abiraterone activity in cancer cells was further investigated in a clinical trial, where the genetic variants of *SLCO2B1* namely the coding rs12422149 (c.935G>A) and the intronic rs1789693 correlated with tissue concentrations of abiraterone. Even if the OATP2B1-935A variant is assumed to exhibit reduced transport function [70], Mostaghel et al. [40] report higher mean tissue abiraterone levels in carriers of the rs12422149 AA/AG genotype as compared to prostate cancer tissue of individuals with homozygosity for the wildtype allele. In contrast, presence of the minor allele of the intronic rs1789693 was associated with lower mean tissue abiraterone levels. Importantly, the authors also showed that higher tissue abiraterone concentrations were associated with decreased serum prostate specific antigen levels and improved pathologic response after radical prostatectomy as compared to lower tissue abiraterone concentrations [40]. In agreement, Hahn et al. [41] corroborated that patients heterozygous for *SLCO2B1* rs12422149 with metastatic castration-resistant prostate cancer receiving first-line abiraterone acetate had improved progression-free survival as compared to homozygous wildtype carriers (GG) (Figure 1). It remains to be determined whether genetic variants of *SLCO2B1* can be used as biomarkers for the response to abiraterone treatment in advanced prostate cancer.

Notably, for other tumor entities such as colorectal neoplasia, liver, or breast cancer, *SLCO2B1* genetic variants have not been associated with a risk of developing cancer [80,131]. Also, investigations on OATP2B1 expression show variable outcome with low predictive character; depending on the cancer type and research group, major differences in OATP2B1 expression levels have been reported even within one tumor entity [116,132,133,134,135,136]. Possible reasons for these overall inconsistencies outlined above might be differences (i) in the choice of the respective control tissue, (ii) in the quality of measure (mRNA or protein), or (iii) or in the presence of different *SLCO2B1* splice variants as previously described for liver and intestine [137].

Even if the abovementioned data on abiraterone suggest a role of OATP2B1 in pharmacokinetics, as there is a change in plasma levels associated to genetic variants of the transporter, we have to note that the role of OATP2B1 in drug absorption and elimination is still unclear, even if expression was shown in organs vital to ADME [70,138]. In the last year, two laboratories independently reported on Oatp2b1 knockout mice [139,140]. The murine orthologue has a high sequence homology with the human OATP2B1 transporter and a similar expression pattern. Chen and coworkers [139] used the Oatp2b1-deficient mouse model to quantify fluvastatin plasma levels in the presence or absence of erlotinib, the latter being assumed to be transported by OATP2B1 but not the OATP1B transporters [141]. Erlotinib is a potent and reversible inhibitor of the epidermal growth factor receptor (EGFR) tyrosine kinase, is orally bioavailable, and is extensively metabolized in the liver predominantly by CYP3A4, and the metabolites are excreted by the biliary system (83%) [141]. Pretreatment with orally administered erlotinib significantly decreased the plasma concentration of fluvastatin in wildtype but not in knockout mice, supporting a role of OATP2B1 in the intestinal handling of fluvastatin. Interestingly, at the same time, the authors did not observe differences in the uptake of fluvastatin in the liver, suggesting that hepatic transporters other than OATP2B1 compensate for Oatp2b1-depletion in mice [139]. Whether deficiency in Oatp2b1 in mice influences the handling of erlotinib remains open. Notably, Bauer et al. [141] reported a significant, however, minor increase in uptake of the radiotracer (^11^C)-erlotinib in A431 cells overexpressing OATP2B1 but not OATP1B1 or OATP1B3, which was inhibited by cyclosporine A or rifampicin. In addition, the authors found the transporter-independent component in cellular accumulation to be considerable. In the same work, PET microdosing of (^11^C)-erlotinib administered intravenously in healthy human subjects showed that erlotinib was taken up by the liver. Pretreatment with peroral therapeutic doses of erlotinib (300 mg) prior to (^11^C)-erlotinib microdosing led to a decrease in liver accumulation with concomitant increase in plasma (^11^C)-erlotinib concentrations [141]. It is not concludingly answered yet whether erlotinib is a substrate of OATP2B1 in vivo. However, given that erlotinib inhibits OATP2B1 much more potently than OATP1B1/3 (IC_50_ 0.03 μM for OATP2B1 as compared to 100/1.1 μM for OATP1B1/3), erlotinib may be used as a selective inhibitor of OATP2B1 [138,142].

## 3. Organic Cation Transporters (OCTs) in Cancer

Considering the electric charge of molecules, there have been attempts to associate the expression of organic cation transporters and the pharmacodynamics of anticancer drugs. Testing various tumor entities for expression of the major OCTs namely OCT1 (*SLC22A1*), OCT2 (*SLC22A2*), and OCT3 (*SLC22A3*) suggests the presence of at least one organic cation transport system in most tumor entities, as excellently summarized by Hermann Koepsell [143]. According to the Cancer Genome Atlas, OCT1 exhibits the highest expression in hepatocellular carcinoma, while OCT2 is most abundant in renal cancer [144]. However, even if there are reports showing significant downregulation of a certain organic cation transporter compared to healthy tissue (summarized in [143]), one may still speculate that antineoplastic drug efficacy of OCT substrates may be influenced by the existing net activity of the organic cation transporting system. It seems noteworthy in this context that, in the healthy organism, OCT1 is predominantly expressed at the sinusoidal membrane of hepatocytes while the majority of OCT2 is found at the basolateral side of proximal tubules, which may explain the observed downregulation in association with dedifferentiation as expected during malignant transformation [145,146]. Irrespective of that, there are data correlating the downregulation of organic cation transporter in cholangiocellular or hepatocellular carcinoma expression with tumor progression or patient survival [147,148]. Whether this is due to reduced cellular uptake of cancer therapeutics remains open in those studies.

One drug class which has been extensively studied in the context of organic cation transporters are the platinum-based drugs [149]. Especially for cisplatin, oxaliplatin and picoplatin transport by OCTs has been shown in vitro [150,151,152,153,154]. Even if reported to be expressed, there is only limited evidence that the expression level of OCTs and especially that of OCT1 and OCT2 is predictive for the efficacy of platinum drugs [149]. In detail, cisplatin was identified as a substrate of OCT1 and OCT2; however, data about its interaction with OCT3 are contradictory [150,151]. Nevertheless, an association between OCT3 expression and treatment response with a longer survival time was observed in patients with head and neck squamous cell cancer. Indeed, patients with high expression of OCT3 benefited from the cisplatin treatment with a longer survival time [52]. Importantly, OCT2 has been linked to cisplatin-induced nephrotoxicity, a major limitation for cisplatin dosing [155]. Here, OCT2, which is highly expressed in the basolateral membrane of the proximal tubular cells (PTCs), is assumed to facilitate PTC entry and therefor exposure of the cells to the toxin [145,146,155]. In Oct1/Oct2 knockout mice, this mechanistic link has clearly been shown [156].

Targeting the transporter to reduce nephrotoxicity was investigated in a study with 123 Chinese cancer patients receiving cisplatin alone or in combination with cimetidine, a known substrate and therefore competitive inhibitor of the OCTs [42]. It has previously been reported that cimetidine does not affect the antitumor efficacy [157,158]. Interestingly, the cimetidine-treated group experienced attenuated cisplatin nephrotoxicity. Furthermore, the rs316019 (*SLC22A2* c.808G>T, p.Ala270Ser) was identified to be nephroprotective, as individuals carrying the wildtype allele were more likely to exhibit an increase of serum creatinine than carriers of the reduced function allele [156]. It is assumed that the reason for this is reduced accumulation of cisplatin in the proximal tubular cells [42,156]. Importantly, neither the knockout mice nor patients with the genetic variants exhibited changes in serum concentration of cisplatin [156]. However, in a recently published work, the correlation between polymorphism and renal damage could not be confirmed and the authors highlighted the need for consistent protocols in order to compare study results in which renal function is measured with biological markers [159]. There is a complex interplay even of uptake transport systems in cisplatin-induced nephrotoxicity. Indeed, Nieskens et al. [160] report that overexpression of the renal organic anion transporters OAT1 and OAT3 influenced the cisplatin-induced toxicity in conditionally immortalized proximal tubule epithelial cells; even if the OATs do not transport cisplatin themselves, there is a reduction in cellular accumulation of the nephrotoxin in OAT-overexpressing cells. However, an in vivo study using Oat1- or Oat3-deficient mice revealed a reduced cisplatin-toxicity measuring marker of acute renal tubular necrosis and histopathologically inspecting the tissues in the knockout animals, suggesting that Oats directly contribute to renal damage. According to the authors, this observation may be linked to the anionic mercapturic acid metabolite of cisplatin (also called NAC-1), which is transported by OAT1 and OAT3, and was most likely not present in the experiments reported by Nieskens et al. [160,161]. Furthermore, in rats, coadministration of the tyrosine kinase inhibitor nilotinib protected the kidney from cisplatin-induced damage [130]. Importantly, nilotinib inhibits OCT2, OAT1, and OAT3 function as shown in vitro, and in this experiment, the cytotoxic effect of cisplatin was not affected [161]. Whether OATs can serve as a target to avoid cisplatin-induced nephrotoxicity has to be confirmed in in vivo experiments. Nevertheless, these findings highlight the contribution of different transporters to drug-induced side effects.

Oxaliplatin is used in the treatment of metastatic colorectal cancer and was identified as a substrate of OCT1 and 2 [152,153]. Interestingly, high expression of OCT2 in metastatic colorectal cancer was linked to long progression-free survival [57]. In another retrospective study, high expressions of OCT2 and OAT2 were identified in 60 and 36% of the cancer specimen of 90 patients with metastatic colorectal cancer treated with the combination 5-FU/leucovorin/oxaliplatin (FOLFOX). In these patients, high expression of OAT2 was associated with a good objective tumor response while high expression of OCT2 was linked to a long progression-free survival. Patients with high expression of both transporters showed the best treatment outcomes [55]. Since oxaliplatin is a substrate of OCT2, the authors assume that the transporter contributes to oxaliplatin uptake into the tumor. OAT2 could be responsible for 5-FU uptake into the tumor as high expression was also shown to be a predictor of good therapy response in colorectal cancer patients [54]; however, in vitro data about the interaction are contentious [162,163]. Finally, for OCT3, there are contradictory in vitro results on its interaction with oxaliplatin [152,153]. Interestingly, mRNA of OCT3 was shown to be increased in colorectal cancer compared to healthy tissue, and in vitro assays revealed that OCT3 could be the responsible transporter for oxaliplatin uptake in colorectal cancer cells [164]. Notably, Le Roy et al. [56] showed that high expression of OCT3 in colorectal cancer was an independent predictor for resistance to FOLFOX therapy; no such correlation was found in their population for OCT1 or OCT2. Consequently, conclusions about OCTs in oxaliplatin therapy remain speculative and further studies are needed.

Finally, it seems noteworthy that there are also studies reporting on the contribution of OATPs to the cellular uptake of platinum-based drugs. OATP1B1 or OATP1B3 ectopically expressed in HEK293 cells enhance cellular uptake of cisplatin, and OATP1B3 also transported carboplatin and oxaliplatin. In line with this, Oatp1b2-deficient mice treated with cisplatin exhibited a significantly reduced platinum liver uptake and increased urinary elimination as compared to wildtype mice [165]. Likewise, conjugates of cisplatin with glycocholate (Bamet-R2) or with ursodeoxycholate (Bamet-UD2), designed as liver-trophic cytostatics to target tumors of the hepatobiliary system, are transported by OATP1B1 and to a lesser extent by OATP1A2, OCT1/2, and sodium/taurocholate cotransporting polypeptide (NTCP) [166]. As seen for many transporter substrates, there seems to be no clear transporter specificity of platinum-based drugs as different transport families are involved in transport of the drug itself, its metabolite, or conjugates.

## 4. Organic Cation Transporter Novel Type (OCTNs)

Organic cation transporter novel (OCTN) type 1 (OCTN1, *SLC22A4*) and OCTN type 2 (OCTN2, *SLC22A5*) are ubiquitously expressed in healthy tissue including the heart, intestine, and kidney [167,168,169,170,171]. Both transporters recognize L-carnitine and are assumed to play a role in systemic and cellular carnitine homeostasis [172,173]. Given that, it is not surprising that both transporters are expressed in most tumors as shown in the Cancer Genome Atlas [144]. However, in 2018, Tschirka and colleagues [174] proposed to rename OCTN1 as “ergothioneine transporter” (ETT) since ergothioneine was the only high affinity substrate of the transporter, suggesting that the name “novel organic cation transporter” is misleading. Nevertheless, there are reports showing transport of other compounds including oxaliplatin, saracatinib, and nucleoside analogs such as cytarabine and gemcitabine [53,175]. These nucleoside analogs are used in the treatment of acute myeloid leukemia (AML), and analysis of primary blast samples revealed that low expression of OCTN1 predicts poor survival [53].

As mentioned before, OCTNs are assumed to be major determinants in carnitine homeostasis. Carnitine is a small molecule essential for the transport of long-chain fatty acids into the mitochondrial matrix, where they get metabolized via β-oxidation [176]. Accordingly, cellular carnitine homeostasis is closely linked to metabolic activity.

In breast cancer, OCTN2 expression has been shown to be significantly higher in estrogen-receptor (ER)-positive than in ER-negative tumor tissue specimens. Its expression is upregulated by estrogen and highly depends on the ER [177]. In glioblastoma in which OCTN2 expression and carnitine concentration were found to be increased compared to healthy brain, high expression of OCTN2 was associated with a significantly worse survival time. In an orthotopic glioblastoma mouse model, the authors showed that the OCTN2 inhibitor meldonium reduced cancer growth [178]. Both OCTNs are also expressed in the intestine, and the genetic variant of *SLC22A4* c.1672TT (rs1050152) was found to increase the risk for colorectal cancer in patients with ulcerative colitis and for sporadic colorectal cancer in younger aged (<55) patients without intestinal bowel disease [169,179]. An increased risk of colorectal cancer was also found in Chinese carriers of the SNP in *SLC22A5*, rs27437 [180].

OCTNs seem to be a factor in tumor progression; however, it remains to be investigated whether modulation of OCTN activity in cancer is a valid approach for growth repression, especially when considering their function in the organism. In this context, it seems noteworthy that there are reports linking the salicylic acid metabolites and their influence on cyclin-dependent kinases to colorectal cancer prevention by aspirin [181,182]. Moreover, a metabolomics analysis of mucosal biopsies obtained from individuals with an approximately three-year treatment with aspirin revealed an influence on carnitine shuttle metabolism [183]. Whether there is a link between aspirin (or its metabolites) and the function of OCTNs in cancer prevention has to be further investigated.

Finally, there are reports linking the OCTN2/carnitine system to drug-induced nephrotoxicity. Patients treated with a platinum-based antineoplastic drug (cisplatin, carboplatin, or oxaliplatin) showed an increased urinary excretion of carnitine [184,185]. This could be explained by a platin drug-associated decrease in PPARα activity, which is a nuclear receptor regulating OCTN2 transcription [186]. However, whether the observed increase in carnitine excretion is indeed linked to diminished uptake of carnitine via OCTN2 or describes the result of tubule necrosis is not yet clarified. In this context, it seems noteworthy that increased urinary excretion of carnitine in patients treated with etoposide was ascribed to direct interaction of the transporter with this drug [187]. Taken together, to further understand the role of OCTN2 in drug-induced nephrotoxicity, more studies are needed.

## 5. The Di- and Tripeptide Transporters (PEPTs)

The human di- and tripeptide transporters PEPT1 (*SLC15A1*) and PEPT2 (*SLC15A2*) facilitate symport of protons with di- and tripeptides. Moreover, PEPT1 and PEPT2 recognize peptide-like drugs such as β-lactam antibiotics, bestatin, 5-aminolevulinic acid (ALA), and the prodrugs valaciclovir and valganciclovir as substrates [188,189,190]. In general, both transporters exhibit a similar portfolio of substrate, however, with differing affinities [191,192]. Both PEPT transporters are expressed in a broad range of different tissues. In the intestine (PEPT1) and the kidney (PEPT1 in the S1 and PEPT2 in the S3 segment of the tubule), PEPTs are the predominant transporters for absorption and reabsorption of dietary digestion products and peptido-mimetic drugs, respectively. In cancer, both PEPT1 and PEPT2 expressions have been reported for several different tumor tissues (bladder, colorectal, and hepatocellular cancer) and cell lines deriving from different tumor entities such as gastric, prostate, pancreatic, and hepatocellular carcinomas [193,194,195,196,197,198]. To date, bestatin and ALA are the only approved compounds on the market that are assumed to use PEPTs as a route through membranes.

In detail, bestatin, which is a dipeptide and natural product of *Streptomyces olivoreticuli*, is orally available and mainly renally eliminated in its unchanged form [199]. Bestatin, currently only approved in Japan, is used as a supplement to chemotherapeutics in acute nonlymphocytic leukemia and squamous cell lung carcinoma [200]. As an inhibitor of aminopeptidases, bestatin was shown to inhibit both endo- and ectopeptidases [201,202,203]. Inhibition of cytosolic aminopeptidases leads to intracellular accumulation of small peptides and a concomitant decrease in the free amino acid pool triggering the so-called amino acid deprivation response, while inhibition of cell surface ectopeptidases, the major target of bestatin on immune cells, induces a complex immune response [202,204]. Hence, for the latter mode of action, bestatin does not need to cross the cellular membrane of tumor cells. Early studies on bestatin transport in intestinal brush-border membranes and in rat renal brush-border vesicles suggested a role of both PEPT but also OCT transporters [199,205,206,207]. Later studies in rats showed that OATs are also involved, indicating that the transport of bestatin is accomplished by several SLC transporters with a so far unknown contribution of each of the transporters [208]. Even if clinical trials suggest efficacy in nonlymphocytic leukemia and squamous cell lung carcinoma, there is currently no conclusive information on bestatin handling by transporters in vivo [209,210].

The second shown substrate of PEPT transporters is ALA, which is a natural non-protein amino acid and the first intermediate during porphyrin biosynthesis. In the cancer field, ALA has gained recognition as a photosensitizer in photodynamic therapy (PDT) as it increases accumulation of photosensitizing protoporphyrin IX (PPIX) in cancer cells as reviewed by Brown et al. [211]. PDT is primarily used to treat skin cancers and precancerous lesions, but its application has recently been extended to Barrett’s oesophagus, intraepithelial neoplasms of the vulva and cervix, bladder cancer, and advanced lung cancer [211,212]. ALA-mediated photodynamic diagnosis (ALA-PDD) and fluorescence-guided surgery (FGS) are additional applications which have been approved for brain surgery to visualize tumor tissue during tumorectomy of malignant gliomas [213]. In recent clinical trials, the use of ALA-PDD was successfully expanded to imaging of bladder and gastric cancers as well as peritoneal metastases of different tumor origin such as ovarian, pancreatic, or gastric cancers [214,215,216]. In terms of pharmacokinetics, it has been shown that, when ALA is administered orally, intravenously, or topically, it is readily taken up by cells with an accumulation primarily in epithelia [217]. Considering that ALA was shown to be a substrate of both PEPT1 and PEPT2 and that uptake in an intestinal cell model was linked to PEPT1, it has been assumed that peptide transporters contribute to in vivo handling [218,219]. However, ALA is also a substrate of other uptake transporters, namely of members of the *SLC6* family of sodium- and chloride-dependent neurotransmitter transporters (taurine transporter (TauT) and GABA transporter 2 (GAT2)). Of note, ALA uptake by GAT2 into peripheral sensory nerve endings has been suggested to account for one of the few adverse side effects of ALA-PDT, namely pain [220]. In summary, the role of the peptide transporters in in vivo handling of ALA and its application on PDT and ALA-PDD remains open.

In the past few years, a couple of preclinical trials have been undertaken to test whether PEPT1/2 could be exploited to target di- or tri-peptide-conjugates to cells. Gong et al. [198] aimed to selectively hit HCC using triple-glycine conjugated doxorubicin. Disappointingly, only a little increase in doxorubicin accumulation in HCC cells as compared to normal liver cells was observed, and thus, limited advantage of the dox-conjugate over conventional doxorubicin treatment on tumor growth and prevention of cardiotoxicity was observed. Moreover, two tyrosine (tyr) dipeptides of *p*-borono-L-phenylalanine (BPA), BPA-Tyr and Tyr-BPA, were synthesized and tested in mice bearing PEPT1-expressing pancreatic cancer AsPC-1 cells. However, the tumor-to-blood ratio of BPA-Tyr in vivo reached only a factor of two and did not meet the requirements for clinical application [221]. In addition to the insufficient intratumoral accumulation, it was shown that BPAs are also substrates of amino acid transporters LAT1, LAT2, and ATB0, +, when overexpressed in *Xenopus laevis* oocytes, indicating that BPAs are not specific for PEPT1 [222]. Finally, two PEPT radiotracers, ^11^C-glycyl-sarcosine and the more sTable 18F-glycyl-sarcosine, were tested for imaging of tumors highly expressing PEPT such as pancreatic, gastric, and prostate tumors [223,224]. In mice bearing subcutaneous AsPC-1 cell-derived tumors, Mitsuoka et al. [223] found an advantageous tumor-to-blood ratio which was increased several fold over the muscle-to-tumor ratio. However, there was also a substantial accumulation in healthy tissues, especially in kidney, liver, lung, and pancreas, putting the selectivity of the radiotracer into question.

Another approach investigated is the inhibition of PEPT. PEPT1 was shown to be upregulated in patients with chronic inflammatory bowel disease who have an increased risk to develop colitis-associated cancer (CAC). The PEPT1 inhibitor Lys-Pro-Val (KPV), a melanocortin-derived tripeptide, was tested in a transgenic PEPT1-overexpressing mouse model of CAC [127]. KPV was found to be effective to reduce colonic inflammation and tumor burden index in a PEPT1-dependent manner with apparently no side effects [127]. The lack of side effects is in line with observations by Hu et al. [225] and Rubio-Aliaga et al. [226] in *Slc15a1-* and *Slc15a2-*deficient mice showing that both knockout strains presented without a pathological phenotype with normal serum clinical chemistry, suggesting that there is redundancy in peptide transport. It might be speculated that also tumor cells circumvent the inhibition of PEPT transporters using redundant transporters upon long-term treatment.

Finally, even if genetic variability for PEPT1 and PEPT2 is rather low, there is a recent report investigating the PEPT2 genetic variant rs2257212 (*SLC15A2* c.1048C>T, p.L350F) as a potential predictor of actinomycin D (act D) pharmacokinetics in pediatric cancer patients [227]. However, this variant was not significantly associated with act D PK. Act D is a polypeptide antibiotic and inhibitor of RNA polymerases used in the treatment of Wilms tumor, rhabdomyosarcoma, and Ewing’s sarcoma [227,228,229,230,231]. Overall, the broad expression of PEPT1 and 2 in healthy organs and the redundancy in transport of “PEPT-specific” drugs, amino acids, and di-/tripeptides challenges the reasonable concept of PEPT-specific targeting or inhibition in tumors.

## 6. Conclusions

Small molecule chemotherapeutics are frequently used in cancer therapy. Hence, it is of major interest to find strategies to improve efficacy and to minimize toxic side effects. In the last two decades, uptake transporters expressed in organs involved in ADME processes were recognized as important determinants in drug disposition. Besides, SLC expression was found in several cancer tissues, where they contribute to cellular accumulation of small molecules.

Even if the data on SLC transporters in cancer significantly contributed to the understanding of the in vivo handling of small molecules, there are currently insufficient data to suggest that uptake transporters could be exploited to achieve tumor specificity in terms of only hitting a certain cell type without influence on healthy cells. This can be attributed to the expression of most SLC transporters in nonmalignant transformed tissues and the fact that most molecules are recognized by multiple transporters. We have focused on uptake transporters, but certainly, there are also efflux transporters, which have to be considered when predicting cellular accumulation. It may be expected that, with more information on transport kinetics in combination with technologies to quantify expression, it may be possible to characterize expression patterns allowing to predict the net transport of a certain compound in a certain tumor. The latter approach is and will be part of an individualized cancer therapy.

## Figures and Tables

**Table 1:** Overview of clinical trials showing selected chemotherapeutics, indication, and association between single nucleotide polymorphisms (SNPs) of SLC transporters and therapy response.

Chemotherapeutic Alone or in Combination (Administration)	Tumor Indication	Transporter	SNP	Response to Treatment (Defined Criteria)	Study Design and Patient Number
Abiraterone (p.o.)	Prostate cancer	OATP2B1	rs12422149 rs1789693	Higher mean drug tissue levels Lower mean drug tissue levels	Clinical trial, 58 patients [40]
	Prostate cancer	OATP2B1	rs12422149	Improved progression-free survival (less increase in PSA and/or radiographic or clinical progression)	Retrospective, 79 patients [41]
Cisplatin (i.v.)	Different malignant solid tumors	OCT2	rs316019	Attenuated nephrotoxicity	Retrospective, 123 patients [42]
Imatinib (i.v.)	GIST	OATP1A2	rs11568563, c.516A>C, p.E172D, OATP1A2*3	No significant changes in imatinib plasma levels	Retrospective, 94 patients [43]
	CML	OCT1, OCTN1, OATP1A2	Combination of SNPs	Major (defined as a 3-log reduction in Bcr-Abl transcript level from a standardized baseline value) and complete (defined as at least a 4-log reduction corresponding to undetectable Bcr-Abl transcript by PCR) molecular response	Retrospective, 189 patients [44]
	CML	OATP1A2	−361GG	Lower clearance	Retrospective, 34 patients [45]
	GIST	OCTN1 OCTN2	rs1050152 rs2631367 rs2631372	Improved time to progression (calculated from the start of imatinib therapy to the date of disease progression documented by a CT scan)	Retrospective, 54 patients [46]
Irinotecan (i.v.)	NSCLC	OATP1B1	521CC, 521TC, −11187GG	Increased exposure of metabolite SN-38	Phase II study, 81 patients [37]
	Metastatic colorectal cancer and advanced/metastatic pancreatic cancer	OATP1B1 OATP1B1	521C c.388A>G	Increased exposure of metabolite SN-38 Longer progression-free survival (elongated time from initiation of irinotecan-based chemotherapy to the date of progression, death, last contact, or censor date)	Retrospective, 127 patients [38]
Methotrexate (p.o./i.v.)	Rheumatoid arthritis	OATP1A2	c.550G>A, p.E184K	Delayed MTX elimination, increased MTX related toxicity	Clinical trial, 60 patients [47,48]
			c.553G>A, p.D185N c.775A>C, p.V255I c.862G>A, p.T259P	No association with MTX related PK or toxicity	
Sorafenib (p.o.)	Different tumors	OATP1B1	rs2306283 rs4149056	Associated with diarrhea and thrombocytopenia	Retrospective, 114 patients [25]
	Hepatocellular carcinoma	PEPT2	rs2257212	Prolonged progression-free survival (Hazard ratios)	Retrospective, 174 patients [34]
Sunitinib (p.o.)	GIST	OCTN2	rs2631367 + rs2631370 + rs2631372	Longer overall survival (Hazard ratios)	Retrospective, 127 patients [49]
		OATP1B3	rs4149117	Longer overall survival (Hazard ratios)	Retrospective, 127 patients [49]
Docetaxel and Doxorubicin (i.v.)	Breast cancer	OATP1A2	rs4762699 rs2857468	High risk for febrile neutropenia	Clinical trial, 155 patients [50]
Cytarabine, Daunorubicin, Etoposide, Mitoxantrone (i.v.)	AML	OATP1B1	rs2291075	Association with event-free and overall survival (Hazard ratios)	Retrospective, 165 pediatric patients [51]

AML, acute myeloid leukemia; CML, chronic myeloid leukemia; NSCLC, non-small cell lung cancer; GIST, gastrointestinal stromal tumor; MTX, methotrexate; PCR, polymerase chain reaction; PK, pharmacokinetic; SNP, single nucleotide polymorphism.

**Table 2:** Overview of clinical trials showing selected chemotherapeutics, indication, and association between SLC transporter expression level and therapy response.

Chemotherapeutic Alone or in Combination (Administration)	Tumor Indication	Transporter, Expression Level (Quality of Measure)	Response to Treatment (Defined Criteria)	Study Design and Patient Number
Cisplatin (i.v.)	Head and neck squamous cell carcinoma	OCT3, high (protein)	Higher 2-year survival rate	Retrospective, 42 patients [52]
Cytarabine (i.v.)	AML	OCTN1, high (mRNA)	Improved event-free and overall survival (Hazard ratios)	Retrospective, 172 patients [53]
5-FU (i.v.)	Colorectal cancer	OAT2, high (protein)	Good histological response	Pre-treatment biopsy, 45 patients [54]
FOLFOX (i.v.)	Metastatic colorectal cancer	OAT2, high (protein)	Good objective tumor response (RECIST)	Retrospective, 90 patients [55]
		OCT2, high (protein)	Longer progression-free survival (less radiological progression or death)	Retrospective, 90 patients [55]
		OCT3, high (protein)	Non-respond to therapy (cancer recurrence within one year)	Retrospective, 31 patients [56]
Irinotecan (i.v.)	Metastatic colorectal cancer and advanced/metastatic pancreatic cancer	OATP1B3, high (protein)	Reduced progression-free survival (reduced time from initiation of irinotecan-based chemotherapy to the date of progression, death, last contact, or censor date)	Prospective, 127 patients [38]
Oxaliplatin (i.v.)	Metastatic colorectal cancer	OCT2, high (protein)	Longer progression-free survival (longer period from the start of first-line chemotherapy until the first evidence of radiological progression or death)	Retrospective, 80 patients [57]
Sorafenib (p.o.)	Hepatocellular carcinoma	OCT1 (mRNA)	Positive association with survival (Hazard ratios)	Retrospective, 60 patients [30]
	Hepatocellular carcinoma	OCT1, expression at the plasma membrane (protein)	Longer overall survival	Retrospective, 39 patients [31]
Anthracycline and taxane (i.v.)	Breast cancer	OATP1A2, OCT6, high expression of both (protein)	Pathologic good and complete response	Retrospective, 124 patients [58]

AML, acute myeloid leukemia; 5-FU, 5-fluorouracil; FOLFOX, 5-FU/leucovorin/oxaliplatin; RECIST, response evaluation criteria for solid tumors.
